# Sir James Paget

**Published:** 1881-10

**Authors:** 


### SIR JAMES PAGET.

Sir James Paget, the president of the recent International Medical Congress, was born in England in 1814. He become a member of the royal college of surgeons in 1836 and an honorary fellow in 1843. He is a member of the Senate of the University of London, and of the Council of the College of Surgeons. He was made a baronet in 1871, and elected president of the college of surgeons in 1875. He is consulting surgeon to St. Bartholomew's hospital, ana surgeon to the Prince of Wales and the Queen. He has published Pathological Catalogue of the Museum of the College of Surgeons; Report on the Use of the Microscope; Lectures on Surgical Pathology and other contributions to surgical literature.

His masterly inaugural address, selections from which will be found elsewhere, is replete with wise and noble utterances. It is characterized by a calm and dignified review of the questions before him. It is pervaded throughout by a spirit of tolerance and gentle charity. It is terse, logical, eloquent and true.

The expression of these lofty sentiments is calculated to increase the influence and to exalt the standing of the medical profession. These broad and catholic declarations constitute a platform of principles upon which any man may feel proud to stand. It is to be hoped that their dissemination "will make the future better and happier than the past," and tend "to the promotion of the whole science, art and charity of medicine."

